# Different Numbers of Long-Pulse 1064-nm Nd-YAG Laser Treatments for Onychomycosis: A Pilot Study

**DOI:** 10.1155/2020/1216907

**Published:** 2020-01-20

**Authors:** Rui-na Zhang, Feng-lin Zhuo, Dong-kun Wang, Li-zhi Ma, Jun-ying Zhao, Lin-feng Li

**Affiliations:** ^1^Department of Dermatology, Beijing Friendship Hospital, Capital Medical University, Beijng 100050, China; ^2^Department of Dermatology, Beijing Evercare Jianxiang Hospital, China; ^3^Department of Dermatology, Chengdu Second People's Hospital, China

## Abstract

**Purpose:**

To examine the benefits of different numbers of 1064-nm Nd-YAG laser treatments in patients with onychomycosis.

**Methods:**

This was a pilot study of patients with onychomycosis who were divided into three groups: four treatment sessions (group A), eight sessions (group B), and 12 sessions (group C). Only infected nails of degrees II–III (Scoring Clinical Index for Onychomycosis) were included. Treatment was given once a week using a long-pulse Nd-YAG 1064-nm laser. Patients were followed at 8, 16, and 24 weeks after the first treatment. Side effects were recorded.

**Results:**

Treatments were completed for 442 nails in 102 patients. The efficacy rates at 8, 16, and 24 weeks were 35.5%, 38.7%, and 37.4% for group A; 31.4%, 41.7%, and 44.0% for group B; and 27.7%, 50.0%, and 55.4% for group C, respectively. There was a significant difference in the efficacy rate at 24 weeks (*P* = 0.016) between groups A and C, but not for groups A vs. B, or for groups B vs. C. No difference in the efficacy rate at 8 or 16 weeks was observed among the three groups. In all three groups, the efficacy was better for degree II nails than for degree III nails (all *P* = 0.016) between groups A and C, but not for groups A vs. B, or for groups B vs. C. No difference in the efficacy rate at 8 or 16 weeks was observed among the three groups. In all three groups, the efficacy was better for degree II nails than for degree III nails (all

**Conclusions:**

The 1064-nm Nd-YAG laser had clinical benefits against onychomycosis. Higher numbers of treatments provided better long-term (24-week) benefits, but had no impact on the short-term outcomes. The efficacy of laser treatment on degree II onychomycosis was better than for degree III.

## 1. Introduction

Onychomycosis is a persistent fungal infection of the nail bed and plate and is most commonly (85–90%) caused by dermatophytes such as *Trichophyton rubrum *[1, 2]. The worldwide incidence of onychomycosis is approximately 3–5% [3, 4] and increases with age [5]. Besides age, the risk factors are male gender, athletes, diabetes, peripheral vascular diseases, and HIV infection [1, 6]. Patients with onychomycosis may experience significant psychosocial problems because of the appearance of the nail, particularly when fingernails are involved [7].

The treatment of onychomycosis is challenging because the infection is embedded within the nail. Common antifungal drugs for external use or oral administration used for the treatment of onychomycosis include fluconazole, itraconazole, and terbinafine [1, 2, 6], but topical antifungal agents barely penetrate the nail plate and do not achieve local therapeutic concentrations, and systemic oral antifungal medications are not applicable for some patients with abnormal liver function or low immune function [8–10]. Therefore, a new treatment approach is needed.

Laser irradiation is an optional modality for treating onychomycosis. The possible indications of laser therapy include resistance to or low efficacy of topical antifungals, relapsing disease, and interactions and adverse events of systemic antifungals [11]. The lasers used for onychomycosis primarily include the carbon dioxide (CO_2_), 870-nm + 930-nm, and Nd-YAG 1064-nm lasers. The CO_2_ laser, which was the earliest method, can directly gasify and degrade tissues, killing the fungi [12]. Fractional CO_2_ laser combined with a topical antifungal agent showed good clinical efficacy for treating onychomycosis and it was suggested that combination therapy had a higher efficacy for treating onychomycosis than did fractional CO_2_ laser alone [13–15]. On the other hand, the CO_2_ laser is no longer used because of pain and trauma. The 870-nm + 930-nm laser is a dual-wavelength near-infrared ray, and involves a thermal effect on fungal metabolism for onychomycosis treatment [16]. A previous study showed that the long-pulse neodymium-doped yttrium aluminum garnet (Nd-YAG) laser at a wavelength of 1064 nm (Beijing Shiji Guangtong Biotechnology Co., Ltd.) used for the treatment of 154 infected nails in 33 patients could cure 52% of the nails [17]. Many studies have confirmed that the 1064-nm Nd-YAG laser is effective against onychomycosis [11, 18–20]. The advantages of this laser include long wavelength, high energy, simple operation, strong penetrability, and no mutagenesis effect on cell DNA [18].

The most optimal number of treatments with the 1064-nm Nd-YAG laser for onychomycosis remains to be validated. Therefore, the aim of the present study was to examine the benefits of different numbers of 1064-nm Nd-YAG laser treatments in patients with onychomycosis. In order to ensure the comparability among groups and to compare the efficacy of different degrees of severity of onychomycosis with the same number of treatment sessions, we used the Scoring Clinical Index of Onychomycosis (SCIO Index) and only included infected nails of degrees II and III.

## 2. Methods

### 2.1. Participants

This was a pilot study of patients with onychomycosis who sought treatments between 2012 and 2015. The study was approved by the ethics committee of our institution and was registered with the Chinese Clinical Trial Registry (ChiCTR-ONC-17012746). All patients signed the informed consent form before participation.

The inclusion criteria were: (1) 18–65 years of age; (2) typical onychomycosis-related symptoms; (3) tested positive on direct microscopy examination; and (4) SCIO index of 6–12, consistent with degrees II–III. Topical antifungal agents were prohibited for 1 month prior to participation, and systemic antifungal agents for 6 months.

The exclusion criteria were: (1) patient dropped out or changed the treatment regimen or follow-up plan; (2) received other antifungal therapy or agents affecting the outcome during the study; (3) showed continuous or semi-continuous nail discoloration (for example, abnormal nail pigmentation caused by the use of topical antifungal therapy such as Castellani solution, nail-coloring dyes or polishes containing magnesium and iron, or occupational exposure to colorants or bitumen, regardless of the therapeutic or cosmetic purpose); (4) used photosensitivity-inducing medications within 6 months; (5) pregnant, (6) subungual hematoma or nevoid formation; or (7) other concomitant onychopathic-induced diseases such as nail-plate psoriasis, lichen planus, or atopic dermatitis. Those who dropped out from the study because of side effects were analyzed for side effects, but not for efficacy.

### 2.2. Grouping and SCIO Index

The patients were randomly divided into three groups: four treatment sessions (group A), eight sessions (group B), and 12 sessions (group C).

Clinical classification, length of involvement, and degree of hyperkeratosis are the three indicators used to determine the severity of onychomycosis, and are directly related to treatment efficacy and number of sessions. Another important factor influencing efficacy is the growth rate of the nail, which mainly depends on age and location of the infected nail. Based on the SCIO index proposed by Sergeev [21] and Hu et al. [22], we semi-quantified and calculated the above five factors, which were divided into three levels and expressed by corresponding score (Table 1). The severity of the infected nails was divided into five degrees: degree I: SCIO < 6, degree II: 6 ≤ SCIO < 9, degree III: 9 ≤ SCIO < 12, degree IV: 12 ≤ SCIO < 15, and degree V: SCIO ≥ 15. Only nails of degrees II–III were included in this study.

### 2.3. Treatment

Treatment was given using a long-pulse Nd-YAG 1064-nm laser (Beijing Shiji Guangtong Biotechnology Co., Ltd.) using the following parameters: 240–348 J/cm^2^, 3-mm spot size, 30-ms pulse duration, and 1-Hz frequency. The laser energy was adjusted based on the thickness of the nail plate. Thicker nails required higher energy. All infected nails in each patient were fully covered for 2 minutes by an incrementally moving laser beam in a spiral pattern, followed by a 2-minute pause, for three cycles. The treatment was performed at 1-week intervals. Patients in group A received four treatment sessions, those in group B received 8 sessions, and those in group C received 12 sessions.

### 2.4. Clinical Effect Assessment

All patients were followed up at 8, 16, and 24 weeks from the first day of treatment. The nails were analyzed and classified into four grades according to a classification modified from Lim et al. [13], as follows: “complete response or cure” (the nail appears fully normal from the proximal nail fold to involved nail), “significant response” (>60% normal-appearing nail compared with the area of the initially infected nail), “moderate response” (20–60% normal-appearing nail), and “no response” (<20% normal-appearing nail). The clinical efficacy rate was defined as the total percentage of nails with complete response and significant response. Side effects were recorded.

### 2.5. Patient Satisfaction

A satisfaction survey was conducted at the end of the study. The satisfaction was classified as: “very satisfied,” “satisfied,” “slightly satisfied,” or “not satisfied.”

### 2.6. Statistical Analysis

SPSS 22.0 (IBM, Armonk, NY, USA) was used for statistical analysis. Continuous variables are presented as mean ± standard deviation, and were analyzed using a one-way analysis of variance (ANOVA) with Tukey's post hoc test. Categorical variables were presented as frequencies (percentage), and were analyzed using Fisher's exact test. Post-hoc *P* < 0.0167 (Bonferonni correction, 0.05/3) was considered statistically different. The McNemar test was used to analyze to compare the variables. Univariable and multivariable logistic regression analyses were performed to analyze the factors associated with efficacy. *P* < 0.05 was considered statistically significant.

## 3. Results

### 3.1. Patients' Characteristics

In group A, seven patients were lost to follow-up and 33 patients were included in the analysis, for a total of 155 nails. In group B, one patient was lost to follow-up and 39 patients were included in the analysis, for a total of 175 nails. In group C, ten patients were lost to follow-up and 30 patients were included in the analysis, for a total of 112 nails. Hence, 442 nails were included in the analysis.

The patients (34 males and 68 females) were 18–65 years of age.  Table 2 presents the characteristics of the patients. There were more nails with <1 mm thickness in group B compared with the two other groups (*P* = 0.008). The patients in group B had more infected nails on the hands, and took less laser energy compared with group A (*P* < 0.05). The clinical types were also different among the three groups (*P* = 0.002). There were no differences among the three groups regarding severity (*P* = 0.908).

### 3.2. Clinical Effect

**Figure 1:**
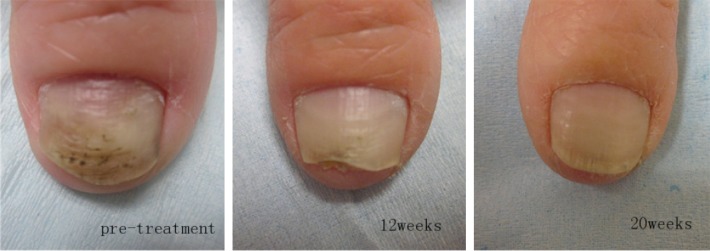
Images before and after four treatment sessions for fingernail onychomycosis.

**Figure 2:**
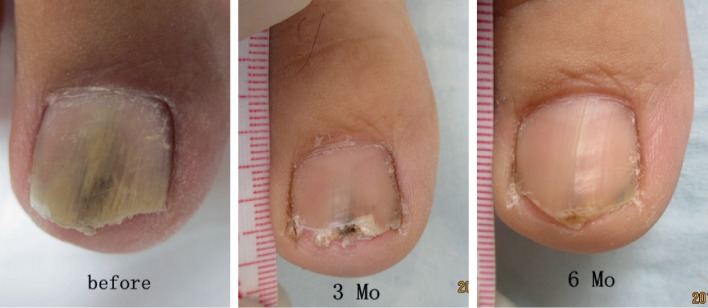
Images before and after eight treatment sessions for toenail onychomycosis.

**Figure 3:**
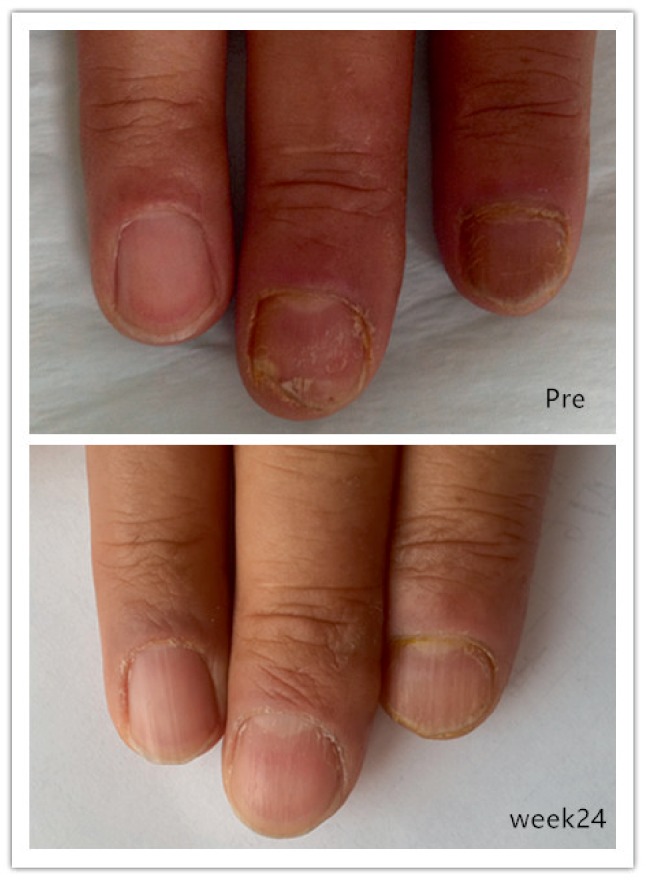
Images before and after 12 treatment sessions for fingernail onychomycosis.

**Figure 4:**
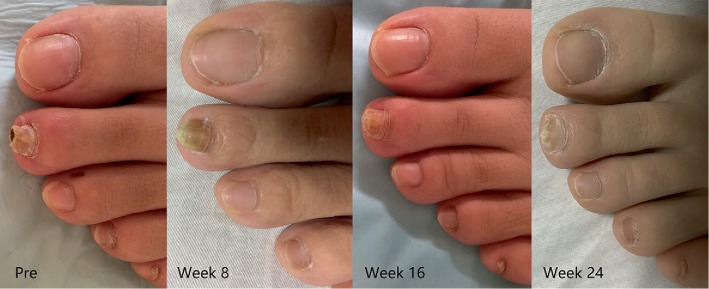
Images before and after 12 treatment sessions for toenail onychomycosis.

The clinical efficacy rates at 8, 16, and 24 weeks were 35.5%, 38.7%, and 37.4% for group A; 31.4%, 41.7%, and 44.0% for group B; and 27.7%, 50.0%, and 55.4% for group C, respectively (Table 3). More nails achieved recovery in group C at week 24 compared with group A (*P* = 0.016), but there were no differences between groups A and B, and between groups B and C. No difference in the efficacy rate at 8 or 16 weeks was observed among the three groups. In terms of severity (Table 4), the effective rate of nails with degree II disease was higher than that of nails with degree III at 8, 16 and 24 weeks in all three groups (all *P* < 0.01). Figures 1–4 present some typical cases.

Multivariable logistic regression analysis for efficacy at 24 weeks supported that group C could achieve a better efficacy (odds ratio [OR] = 2.589, 95% confidence interval [CI]: 1.342–4.994, *P* = 0.005), while degree III was a risk factor (OR = 0.107, 95%CI: 0.052–0.219, *P* < 0.001) (Table 5). In addition, age and nail thickness were independently associated with efficacy.

### 3.3. Satisfaction

In group A, four patients were very satisfied (12.1%), six were satisfied (18.2%), 16 were slightly satisfied (48.5%), and seven were dissatisfied (21.2%). In group B, eight patients were very satisfied (20.5%), 19 were satisfied (48.7%), seven were slightly satisfied (18.0%), and five were dissatisfied (12.8%). In group C, 10 patients were very satisfied (30.3%), five were satisfied (16.7%), 10 slightly were satisfied (30.3%), and five were dissatisfied (16.7%) (Table 6). Satisfaction was higher for group B compared with group A (*P* = 0.025), without difference between groups A and C (*P* = 0.240), and between groups B and C (*P* = 0.065).

### 3.4. Safety

No side effects were experienced by the 102 patients.

## 4. Discussion

The 1064-nm long-pulse Nd-YAG laser can be used to treat onychomycosis, but previous studies had small sample sizes and did not examine the impact of the number of treatments on the outcomes. In our study, we enrolled more subjects (102 patients with 442 effected nails) and included different numbers of treatment. We found that efficacy at 24 weeks was significantly in group C vs. group A, but not between groups A and B, or between groups B and C, as supported by the multivariable analysis. There were no differences among the three groups at 8 and 16 weeks. The results suggest that higher numbers of treatments provided better long-term (24-week) benefits, but the number of treatments had no impact on the short-term outcomes (8 and 16 weeks). In this study, we used the SCIO for evaluating the severity of onychomycosis. The SCIO index was first proposed by Sergeev et al. [21] and subsequently simplified by Hu et al. [22]. We found that the efficacy rate was negatively correlated with the SCIO index. The efficacy was higher in nails of degree II (SCIO 6–8) than in degree III nails (SCIO 9–11) at 8, 16, and 24 weeks and in all three groups (all *P* < 0.05). Hence, we concluded that the long-pulse 1064-nm Nd-YAG laser is more suitable for the treatment of degree II than for degree III onychomycosis. More severely infected nails (degree III) may need a combination of oral drugs or other treatments to improve the efficacy rate of the laser. The SCIO index may have a role in planning laser treatment for onychomycosis.

Regarding recurrence, the numbers of nails with recurrence in groups A and B were 11 (7.1%) and 16 (9.1%) at week 24, respectively, compared with two (1.8%) effected nails in group C. It was presumed that the fungi might have been partially killed or inhibited after laser irradiation, but that their reproductive capacity was gradually restored with time. Therefore, some infected nails that had improved early in the study returned to baseline when treatment ended. Second, the longer the treatment, the lower the recurrence rate. According to the present study, Group C was the most effective, with a rate of 55.4% at 24 weeks. Therefore, it may be hypothesized that the number of treatments should be extended as far as possible and, if necessary, treatments should be given until the infected nail is completely replaced by the new nail. A previous study with the CO_2_ laser showed no recurrence at 3 months [13], but the observation period was shorter than in the present study (12 vs. 24 weeks). Using the *Q*-switched Nd-YAG 1064-nm/532-nm laser, Kalokasidis et al. [5] showed a cure rate of 95.4% at 3 months. Wanitphakdeedecha et al. [19] showed cure rates of 63.5%, 57.7%, and 51.9% at 1, 3, and 6 months, indicating that recurrence rates were higher than in the present study. Of course, differences among treatment protocols might be responsible, at least in part, for the discrepancies observed among studies.

The response rates in this study were lower than those of oral drugs, which show cure rates of 76% for terbinafine, 59–63% for itraconazole, and 48% for fluconazole [1, 2, 6]. The possible reasons could be that the laser is most effective only during heat stimulation. When heat stimulation is interrupted, the fungi gradually grow again, interrupting clinical improvements and the efficacy rate or even leading to relapse. Antifungal agents such as terbinafine and itraconazole have high affinity to keratin, resulting in higher concentrations in the nails than in other body compartments. These agents can remain in the nails for 6–9 months after drug discontinuation [23]. The combination of drugs with the 1064-nm Nd-YAG laser should be explored [22]. Indeed, the mechanism of action of the laser is different from that of the drugs. The long-pulse Nd-YAG 1064-nm laser is characterized by a long wavelength, and the light energy penetrates the nail plate and reaches the nail bed. Chromophores in the walls of fungal cells can absorb this light energy and transform it into heat to damage the cell wall, resulting in fungal apoptosis [24, 25]. The laser also increases the temperature (to approximately 40 °C)of the nail plate at the treatment site [16]. The fungi are damaged by repeated heat stimulation and the fungal mitochondria produce excess reactive oxygen species that overwhelm the protective capacity of the fungal cells, resulting in cell/fungal apoptosis and even death. The main advantage of antifungal agents is the maintenance of the effect even after the end of treatment. On the other hand, systemic drugs are sometimes contraindicated in some patients (e.g., those with liver dysfunction), while topical drugs are associated with a compliance issue [26]. Lasers could be used as an alternative in those cases and in patients who do not want to take medicine. In addition, combination treatments (laser and drugs) should be explored in the future.

Regarding the satisfaction survey, only four (12.1%) out of 33 people were very satisfied and six (18.2%) were satisfied in group A (four sessions), when the survey was conducted at 24 weeks. In group B, most patients were satisfied with treatment (“very satisfied” and “satisfied” represented 20.5% and 48.7%, respectively). Interestingly, although the effective rate was the highest in group C (12 sessions), the satisfaction degree (“very satisfied” and “satisfied” were 30.3% and 17.6%, respectively) was not higher than in group B. This could be due, at least of part, to a higher treatment burden in terms of number of visits to the hospital. This may be a disadvantage of laser treatment for onychomycosis. Treatment regimens need to be optimized to improve therapeutic effects and patient satisfaction.

This study has limitations. A mycology assessment was not conducted and the follow-up was relatively short.

## 5. Conclusions

The 1064-nm Nd-YAG laser has clinical benefits against onychomycosis, without any side effect. Higher numbers of treatments provide better benefits at 24 weeks, but the number of treatments had no impact on the short-term outcomes (8 and 16 weeks). The efficacy of laser treatment on degree II onychomycosis was better than for degree III. SCIO may be used for planning treatment.

## Figures and Tables

**Table 1:** Simplified scoring clinical index of onychomycosis.

Variables	Scoring		
	1	2	3
Clinical classification	WSO	DLSO	PSO or TDO
Length of involvement	<1/3	1/3–2/3	>2/3
Degree of hyperkeratosis	<1 mm	1-2 mm	>2 mm
Age of patients (years)	15–24	25–60	61–75
Location of infected nail	2–4 fingernails	Thumbnail or 2–4 toenails	First toenail

WSO: white superficial onychomycosis; DLSO: distal lateral subnail onychomycosis: PSO: proximal subnail onychomycosis; TDO: total dystrophy onychomycosis.

**Table 2:** Patients' characteristics.

Variable	Group A (*N* = 33)	Group B (*N* = 39)	Group C (*N* = 30)	*P*
Age (years)	50.40 ± 12.5	47.00 ± 10.89	49.03 ± 9.96	0.308
Duration of disease (years)	2.73 ± 1.22	2.39 ± 1.09	2.80 ± 1.51	0.338
Gender, *n* (%)				0.949
Male	11 (33.3%)	13 (33.3%)	11 (36.7%)	
Female	22 (66.7%)	26 (66.7%)	19 (63.3%)	
Total number of infected nails, *n*	155	175	112	
Nail thickness (mm)				0.008
<1	123 (79.4%)	159 (90.9%)^a^	92 (82.1%)^b^	
1–2	32 (20.6%)	16 (9.1%)^a^	20 (17.9%)^b^	
Mean laser energy (J/cm^2^)	292.05 ± 13.83	283.14 ± 12.96^a^	289.77 ± 18.13	<0.001
Location of infected nails, *n* (%)				0.014
Fingernail	17 (11.0%)	39 (22.3%)^a^	15 (13.4%)	
Toenail	138 (89.0%)	136 (77.7%)^a^	97 (86.6%)	
Severity of infected nails, *n* (%)				0.908
II	75 (48.4%)	88 (50.3%)	57 (50.9%)	
III	80 (51.6%)	87 (49.7%)	55 (49.1%)	
Clinical type of onychomycosis, *n* (%)				0.002
DLSO	108 (69.7%)	103 (58.9%)^a^	79 (70.5%)^a,b^	
WSO	20 (12.9%)	26 (14.9%)^a^	3 (2.7%)^a,b^	
PSO	9 (5.8%)	6 (3.4%)^a^	3 (2.7%)^a,b^	
TDO	18 (11.6%)	40 (22.9%)^a^	27 (24.1%)^a,b^	

Group A: four sessions; Group B: eight sessions; Group C: 12 sessions. DLSO: distal lateral subnail onychomycosis; WSO: white superficial onychomycosis; PSO: proximal subnail onychomycosis; TDO: total dystrophy onychomycosis. ^a^*P* < 0.05, vs. group A; ^b^*P* < 0.05, vs. group B.

**Table 3:** Efficacy rates among the different groups.

Clinical efficacy rate			Group A (*n* = 155)	Group B (*n* = 175)	Group C (*n* = 112)	*P*
Week 8	Total		55 (35.5%)	55 (31.4%)	31 (27.7%)	0.095
	Clinical type					
		DLSO	46 (42.6%)	39 (37.9%)	27 (34.2%)	
		WSO	6 (30.0%)	9 (34.6%)	1 (33.3%)	
		PSO	1 (11.1%)	2 (33.3%)	0%	
		TDO	2 (11.1%)	5 (12.5%)	3 (11.1%)	
	Severity of infected nails					
		II	47 (62.7%)	46 (52.3%)	28 (49.1%)	
		III	8 (10.0%)	9 (10.3%)	3 (5.5%)	
	Location of infected nails					
		Fingernail	4 (23.5%)	9 (23.1%)	4 (26.7%)	
		Toenail	51 (37.0%)	46 (33.8%)	27 (27.8%)	

Week 16	Total		60 (38.7%)	73 (41.7%)	56 (50.0%)	0.172
	Clinical type					
		DLSO	51 (47.2%)	55 (53.4%)	42 (53.2%)	
		WSO	6 (30.0%)	11 (42.3%)	3 (100.0%)	
		PSO	1 (11.1%)	3 (50.0%)	1 (33.3%)	
		TDO	2 (11.1%)	4 (10.0%)	10 (37.0%)	
	Severity of infected nails					
		II	53 (70.7%)	62 (70.5%)	37 (64.9%)	
		III	7 (8.8%)	11 (12.6%)	19 (34.6%)	
	Location of infected nails					
		Fingernail	7 (41.2%)	13 (33.3%)	7 (46.7%)	
		Toenail	53 (38.4%)	60 (44.1%)	49 (50.5%)	

Week 24	Total		58 (37.4%)	77 (44.0%)	62 (55.4%)^∗^	0.014
	Clinical type					
		DLSO	51 (47.2%)	54 (52.4%)	45 (57.0%)	
		WSO	6 (30.0%)	11 (42.3%)	3 (100.0%)	
		PSO	0	2 (33.3%)	1 (33.3%)	
		TDO	1 (5.6%)	10 (25.0%)	13 (48.2%)	
	Severity of infected nails					
		II	54 (72.0%)	58 (65.9%)	41 (71.9%)	
		III	4 (5.0%)	19 (21.8%)	21 (38.2%)	
	Location of infected nails					
		Fingernail	7 (41.2%)	13 (33.3%)	8 (53.3%)	
		Toenail	51 (37.0%)	64 (47.1%)	54 (55.7%)	

Group A: four sessions; Group B: eight sessions; Group C: 12 sessions. DLSO: distal lateral subnail onychomycosis; WSO: white superficial onychomycosis; PSO: proximal subnail onychomycosis; TDO: total dystrophy onychomycosis. The clinical efficacy rate was defined as the total percentage of nails with complete response and significant response. “Complete response or cure” was defined as fully normal appearing nail measured from the proximal nail fold to involved nail; “significant response” was defined as >60% normal-appearing nail compared with the area of the initially infected nail; “moderate response” was defined as 20–60% normal-appearing nail; and “no response” was defined as <20% normal-appearing nail. ^∗^*P* < 0.0167, group A vs. group C (Bonferroni correction: 0.05/3). There were no significant differences for group A vs. group B and group B vs. group C.

**Table 4:** Efficacy rates according to severity of onychomycosis in each group at different time points.

Group	Cases, *n*	Efficacy rate, *n* (%)		
		Week 8	Week 16	Week 24
Group A				
II	75	47 (62.7%)	53 (70.7%)	54 (72.0%)
III	80	8 (10.0%)	7 (8.8%)	4 (5.0%)
*P*		<0.001	<0.001	<0.001
Group B				
II	88	46 (52.3%)	62 (70.5%)	58 (65.9%)
III	87	9 (10.3%)	11 (12.6%)	19 (21.8%)
*P*		<0.001	<0.001	<0.001
Group C				
II	57	28 (49.1%)	37 (64.9%)	41 (71.9%)
III	55	3 (5.5%)	19 (34.6%)	21 (38.2%)
*P*		<0.001	0.001	<0.001

Group A: four sessions; Group B: eight sessions; Group C: 12 sessions. The clinical efficacy rate was defined as the total percentage of nails with complete response and significant response. “Complete response or cure” was defined as fully normal appearing nail measured from the proximal nail fold to involved nail; “significant response” was defined as >60% normal-appearing nail compared with the area of the initially infected nail; “moderate response” was defined as 20–60% normal-appearing nail; and “no response” was defined as <20% normal-appearing nail.

**Table 5:** Univariable and multivariable logistic regression analysis for efficacy rate at 24 weeks.

Variable	Univariable logistic regression		Multivariable logistic regression	
	OR (95%CI)	*P*	OR (95%CI)	*P*
Treatment group				
Group A	Reference			
Group B	1.314 (0.845, 2.043)	0.225	1.009 (0.564, 1.805)	0.976
Group C	2.074 (1.265, 3.401)	0.004	2.589 (1.342, 4.994)	0.005
Age (years)	0.919 (0.896, 0.941)	<0.001	0.900 (0.870, 0.931)	<0.001
Duration of disease (years)	0.789 (0.671, 0.927)	0.004	1.114 (0.883, 1.406)	0.363
Gender				
Male	Reference			
Female	1.320 (0.882, 1.974)	0.177		
Nail thickness (mm)				
<1	Reference			
1–2	0.075 (0.030, 0.191)	<0.001	0.24 (0.079, 0.734)	0.012
Mean laser energy (J/cm^2^)	0.979 (0.967, 0.992)	0.001	0.998 (0.981, 1.015)	0.836
Location of infected nails				
Fingernail	Reference			
Toenail	1.285 (0.765, 2.157)	0.343		
Severity of infected nails				
II	Reference			
III	0.108 (0.070, 0.168)	<0.001	0.107 (0.052, 0.219)	<0.001
Clinical type of onychomycosis		<0.001		0.734
DLSO	Reference			
WSO	0.644 (0.348, 1.190)	0.160	0.968 (0.441, 2.127)	0.936
PSO	0.187 (0.053, 0.659)	0.009	0.575 (0.124, 2.674)	0.480
TDO	0.367 (0.217, 0.621)	<0.001	1.304 (0.576, 2.955)	0.525

Group A: four sessions; Group B: eight sessions; Group C: 12 sessions. OR: odds ratio; CI: confidence interval; DLSO: distal lateral subnail onychomycosis; WSO: white superficial onychomycosis; PSO: proximal subnail onychomycosis; TDO: total dystrophy onychomycosis.

**Table 6:** Satisfaction survey.

Satisfaction	Group A (*N* = 33)	Group B (*N* = 39)	Group C (*N* = 30)	*P*
Very satisfied	4 (12.1%)	8 (20.5%)	10 (30.3%)	0.010
Satisfied	6 (18.2%)	19 (48.7%)	5 (16.7%)	
Slightly satisfied	16 (48.5%)	7 (18.0%)	10 (30.3%)	
Not satisfied	7 (21.2%)	5 (12.8%)	5 (16.7%)	

Group A: four sessions; Group B: eight sessions; Group C: 12 sessions. Group A vs. Group B, *P* = 0.025; Group A vs. Group C, *P* = 0.240; Group B vs. Group C, *P* = 0.065.

## Data Availability

The datasets generated and analyzed during the present study are available from the corresponding author on reasonable request.
